# Understanding Real-World Treatment Patterns and Clinical Outcomes among Metastatic Melanoma Patients in Alberta, Canada

**DOI:** 10.3390/curroncol30040317

**Published:** 2023-04-13

**Authors:** Dylan E. O’Sullivan, Devon J. Boyne, Priyanka Gogna, Darren R. Brenner, Winson Y. Cheung

**Affiliations:** 1 Department of Cancer Epidemiology and Prevention Research, Alberta Health Services, Calgary, AB T2S 3C3, Canada; 2 Department of Oncology, University of Calgary, Calgary, AB T2N 4N2, Canada; 3 Department of Community Health Sciences, University of Calgary, Calgary, AB T2N 4N2, Canada; 4 Oncology Outcomes Initiative, University of Calgary, Calgary, AB T2N 4N2, Canada; 5 Department of Public Health Sciences, Queen’s University, Kingston, ON K7L 3N6, Canada

**Keywords:** metastatic melanoma, population-based study, immunotherapy, targeted therapy, lines of therapy, overall survival, real-world evidence

## Abstract

Immunotherapy and targeted therapies have been shown to considerably improve long-term survival outcomes in metastatic melanoma patients. Real-world evidence on the uptake of novel therapies and outcomes for this patient population in Canada are limited. We conducted a population-based retrospective cohort study of all metastatic melanoma patients diagnosed in Alberta, Canada (2015–2018) using electronic medical records and administrative data. Information on *BRAF* testing for patients diagnosed in 2017 or 2018 was obtained through chart abstraction. In total, 434 metastatic melanoma patients were included, of which 110 (25.3%) were de novo metastatic cases. The median age at diagnosis was 66 years (IQR: 57–76) and 70.0% were men. *BRAF* testing was completed for the majority of patients (88.7%). Among all patients, 60.4%, 19.1%, and 6.0% initiated first-line, second-line, and third-line systemic therapy. The most common therapies were anti-PD-1 and targeted therapies. The two-year survival probability from first-line therapy, second-line therapy, and third-line therapy was 0.50 (95% CI: 0.44–0.57), 0.26 (95% CI: 0.17–0.40), and 0.14 (95% CI: 0.40–0.46), respectively. In the first-line setting, survival was highest for patients that received ipilimumab or ipilimumab plus nivolumab, while targeted therapy had the highest survival in the second-line setting. This study indicates that novel therapies improve survival in the real world but a considerable proportion of patients do not receive treatment with systemic therapy.

## 1. Introduction

Malignant melanoma is an aggressive form of skin cancer that arises in melanocyte cells in response to the production of melanin [1,2]. Although the skin is the most commonly affected organ, melanoma can arise in the eyes, mucosa, vulva, or intestines [3,4]. Melanoma is the seventh most common cancer in Canada with 9000 cases projected to have occurred in 2022 [5]. The incidence of cutaneous melanoma has been steadily increasing in Canada [6] and is projected to continue to increase in the future [7].

Diagnostic features of melanoma can be obvious based on a surface-level examination or may require the use of optical tools, with the following features typically considered for diagnosis: asymmetry, border irregularity, color variation, diameter (>6 mm), and evolving (ABCDE) [8]. Most cases of melanoma are diagnosed at an early stage, which has been attributed to increased awareness of the malignancy [9]. At this stage, surgical excision may be curative [10]. However, a small proportion of patients have metastatic disease at presentation, and some develop metastases after their initial definitive treatment [10,11,12]. Currently, many therapeutic options exist for the treatment of melanoma, including surgical resection, chemotherapy, immunotherapy, and targeted therapy [13,14]. Therapeutic strategies may include single or combined therapies [13,14]. 

Current research has led to the development of immunotherapy using checkpoint inhibitors (the anti-programmed cell death 1 (PD-1) antibodies and the anti-cytotoxic T lymphocyte-associated protein 4 (CTLA-4) antibody) and targeted therapy (inhibition of the *BRAF* and/or *MEK* genes) [13,15]. Both immunotherapy and targeted therapy prolong progression-free and overall survival compared with chemotherapy [13,15]. However, there may be acquired resistance to targeted therapies and immunotherapy with studies indicating overall survival just above 50% for checkpoint inhibitors [13,15,16]. 

Recent studies have sought to quantify the rates of advanced malignant melanoma and associated patient characteristics and outcomes based on real-world evidence [17,18,19,20,21,22,23]. Despite some well-documented epidemiological studies, the prevalence, treatment patterns, and outcomes among Canadians require additional investigation. Furthermore, real-world treatment patterns, treatment rates, and outcomes across key subpopulations and those proceeding from one line of therapy to another are lacking. With the melanoma treatment landscape changing rapidly, it is important to understand current treatment patterns and outcomes within this population and its subpopulations to better understand optimal therapies and the potential impact of novel therapies.

## 2. Materials and Methods

### 2.1. Study Design

This study was a retrospective longitudinal cohort study that leveraged real-world, population-level data in Alberta, Canada. The database includes Alberta’s integrated provincial healthcare system, cancer registry, electronic health records, and lab and pathology results. Additional covariates are captured through the hospitalization discharge abstract database, physician billing claims, and the national ambulatory care reporting system databases maintained by the Alberta Government. The database covers 17 cancer centers (2 tertiary centers, 4 regional and 11 community centers), covering 4.5 million residents of Alberta.

The study population included all patients over the age of 18 with a de novo diagnosis of metastatic melanoma (Stage IV) or diagnosis of recurrent metastatic melanoma after progression from early stages (Stage I, II, and III) between January 2015 to December 2018. Patients that presented with early-stage disease were classified as recurrent cases if they received two or more cycles of systemic therapy more than one year after the date of the primary treatment, had initiated radiation therapy more than one year after the date of primary treatment, or had a death due to melanoma [24]. Patients were followed until death or until December 2019, whichever came first.

### 2.2. Study Measures and Outcomes

Study measures from administrative databases evaluated included patient demographics and clinical characteristics, treatment sequence patterns, and clinical outcomes such as overall survival and cancer-specific survival. Baseline characteristics were only estimated for de novo metastatic melanoma since the exact date of recurrence and information at the time of recurrence are not captured within the administrative datasets for recurrent cases. Baseline demographics and clinical characteristics in this study included age, sex, urban/rural residence, measures of socioeconomic status (neighborhood level income and education), Charlson Comorbidity Index (CCI), specific comorbidities, number of metastases, site of metastases, and treatment center. Due to small cell sizes, treatments were categorized as (1) ipilimumab monotherapy or ipilimumab plus nivolumab; (2) PD-1 therapy; or (3) targeted therapy (BRAF monotherapy or BRAF/MEK combination therapy) for all analyses.

### 2.3. BRAF Testing 

Information on *BRAF* testing for patients diagnosed with metastatic melanoma in 2017 or 2018 was obtained through chart abstraction. For each patient, the following were obtained: if the patient had a *BRAF* test, the methods used for the test, the date of the *BRAF* test, and the date of receipt of the results. To compare the distribution of the baseline characteristics by *BRAF* status, *p*-values corresponding to t-tests for continuous variables and chi-square tests for categorical variables are presented as standardized mean differences (SMD), in which values > 0.1 are indicative of an imbalance [25,26]. Unlike *p*-values, the SMD is not dependent on the sample size and quantifies the magnitude of imbalance between groups [25].

### 2.4. Statistical Analyses

Continuous study measures were reported descriptively with median and interquartile range (IQR). Frequencies and percentages were used to report categorical measures of interest. All cell counts with fewer than 10 patients were suppressed (reported as <10 in tables) due to data privacy regulations. A Sankey diagram was generated to depict the relative sample sizes and proportions of patients receiving different therapies along the treatment trajectory from first-line to second-line. With respect to treatment duration, time on therapy was estimated as the time from initiation to the last systemic therapy cycle plus 21 days (typical duration of a cycle of systemic therapy) or until the initiation of the subsequent line of systemic therapy, whichever came first (patients were censored at death or end of study). The median time on therapy was estimated with the Kaplan–Meier method. Survival curves and median time-to-event were estimated via the Kaplan–Meier method for overall survival (OS), 2 year survival, and 5 year survival. Log-rank tests were used to estimate differences in survival curves between disease types or treatments.

## 3. Results

### 3.1. Patient Characteristics

A total of 434 metastatic melanoma patients were included in this study with 110 (25.3%) patients diagnosed with de novo metastatic melanoma and 324 (74.7%) early-stage patients with evidence of a recurrence of metastatic melanoma. Baseline patient demographics and clinical characteristics for de novo patients are presented in Table 1. The median age of de novo patients in this study was 65.96 (IQR = 57.31, 76.31) and there was a higher proportion of men (70.0%). Of the de novo patients, 82.7% had an urban residence. The median neighborhood household income was $37,344.46 (IQR = $31,721.02, $45,489.21) and the median proportion of neighborhood residents who achieved high school education or greater was 0.80 (IQR = 0.73, 0.84). The majority of patients had no comorbidities (74.5%), with cardiovascular disease and diabetes present in 10% of patients. The majority of patients had one or two metastases at diagnosis (67.3%), and the most common sites were pulmonary/pleura (53.6%), lymph nodes (41.8%), brain (28.2%), hepatic (24.5%), and osseous (20.9%).

### 3.2. BRAF Testing

Of the 62 metastatic melanoma patients (diagnosed between 2017 and 2018) with charts available for *BRAF* testing abstraction, 55 (88.7%) were tested for a *BRAF* mutation. The majority of tests were conducted using the Qiagen *BRAF* RGQ PCR kit (81.8%), while 7.2% were conducted using next-generation sequencing, and 10.9% were conducted using other methods. Among the 55 metastatic melanoma patients who were tested, 23 (41.8%) were *BRAF*-mutant and 32 (58.2%) were *BRAF*-wildtype. The median time for test results was 11.0 days (IQR = 10.0 days; range = 2.0 to 49.0 days). Demographic characteristics of mutant and wildtype *BRAF* patients are presented in Table 2. There was some indication that *BRAF*-mutant patients were younger than *BRAF*-wildtype (*p*-value = 0.063; SMD = 0.562).

### 3.3. Treatment Patterns

Among all metastatic melanoma patients in this study, 262 (60.4%) initiated first-line, 83 (19.1%) initiated second-line, and 26 (6.0%) initiated third-line systemic therapy (Table 2). Out of the 54 patients with data available on their *BRAF* mutation status, 45 (83.3%) initiated first-line systemic therapy and 16 (29.6%) initiated second-line therapy. Among all patients in this study (regardless of BRAF mutation status) that initiated first-line therapy, 43.9% received anti-PD-1 therapies, 32.1% received a targeted therapy and 24.0% received ipilimumab or ipilimumab plus nivolumab. Due to small cell counts, quantitative results for the type of regimen by *BRAF* status could not be presented. Qualitatively, the majority of *BRAF*-wildtype patients were treated with anti-PD-1 therapies, and the majority of *BRAF*-mutant patients were treated with a targeted therapy in the first-line setting. There was not a significant difference in time to *BRAF* testing between patients that received targeted therapy and patients that received anti-PD-1 therapies (mean difference = −3.2 (95% CI: −17.9 to 11.6) days). The median time from diagnosis to first-line systemic therapy was 7.0 weeks (IQR = 7.4) for targeted therapy, 9.1 weeks (IQR = 11.1) for PD-1, and 9.3 weeks (IQR = 3.8) for ipilimumab or ipilimumab plus nivolumab. The median time on first-line therapy was 31.0 weeks for anti-PD-1, 26.7 weeks for targeted therapy, and 13.1 weeks for ipilimumab or ipilimumab plus nivolumab.

In the second-line setting (regardless of BRAF mutation status), a similar proportion of patients received anti-PD-1 (39.8%) and targeted therapy (38.6%), while fewer patients received ipilimumab or ipilimumab plus nivolumab (19.3%). The median time on second-line therapy was 19.4 weeks for targeted therapy, 18.0 weeks for anti-PD-1, and 5.9 weeks for ipilimumab or ipilimumab plus nivolumab. The majority of patients who initiated third-line therapy received anti-PD-1 (46.2%) or targeted therapy (38.5%). The median time on third-line therapy was 19.0 weeks for anti-PD-1 and 16.0 weeks for targeted therapy.

**Figure 1:**
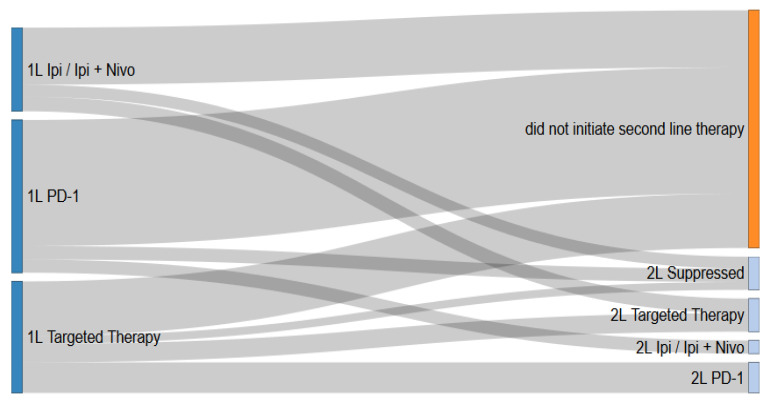
Treatment patterns of metastatic melanoma patients in Alberta, Canada from first-line to second-line therapy.

The sequencing of first-line to second-line therapy is presented in Figure 1. A higher proportion of patients that received a targeted therapy in first-line initiated second-line therapy (51.2%), compared to patients that received anti-PD-1 therapy (31.8%) or patients that received ipilimumab or ipilimumab plus nivolumab (17.1%) in first-line. Patients that received ipilimumab or ipilimumab plus nivolumab for first-line therapy primarily received a targeted therapy for second-line therapy (55%), while for patients that received anti-PD-1 for first-line therapy, ipilimumab or ipilimumab plus nivolumab was the most common second-line therapy (50%). Among patients that received a targeted therapy in first-line, the majority of patients received anti-PD-1 (53.4%) or a targeted therapy (32.6%) in the second-line setting.

### 3.4. Survival Outcomes

**Figure 2:**
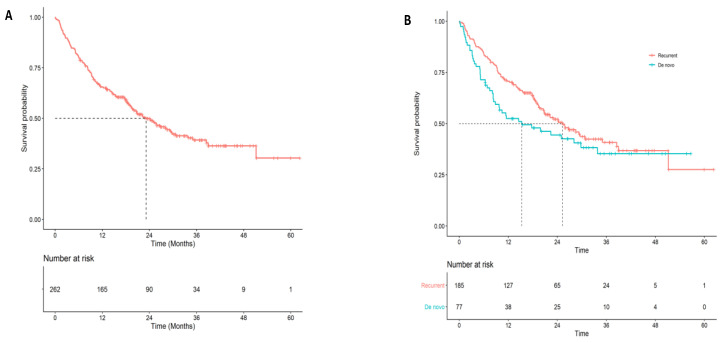
Overall survival for first-line systemic therapy overall and by disease type (**A**) Overall survival for all patients that initiated first-line systemic therapy. (**B**) Overall survival for first-line systemic therapy by disease type (de novo vs. recurrent).

Among de novo cases, 2 year survival was considerably higher for patients that were treated with systemic therapy (chemotherapy, immunotherapy, and targeted therapy) (0.48; 95% CI: 0.38–0.61) compared to patients that were not (0.30; 95% CI: 0.17–0.51; *p* = 0.029). The two-year survival probability from first-line therapy, second-line therapy, and third-line therapy was 0.50 (95% CI: 0.44–0.57; Figure 2A), 0.26 (95% CI: 0.17–0.40), and 0.14 (95% CI: 0.40–0.46), respectively. Among patients treated with first-line systemic therapy, there was some indication that recurrent patients had better overall survival than de novo patients (*p* = 0.13; Figure 2B; Table S1). 

**Figure 3:**
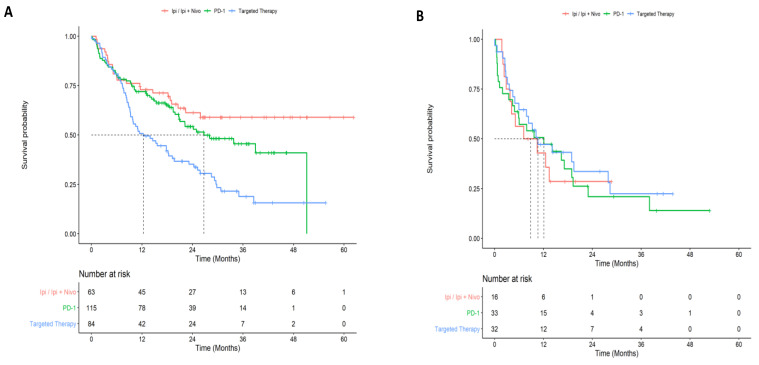
Overall survival by treatment for first and second-line systemic therapy. (**A**) First-line overall survival by type of systemic therapy regimen. (**B**) Second-line overall survival by type of systemic therapy regimen.

**Figure 4:**
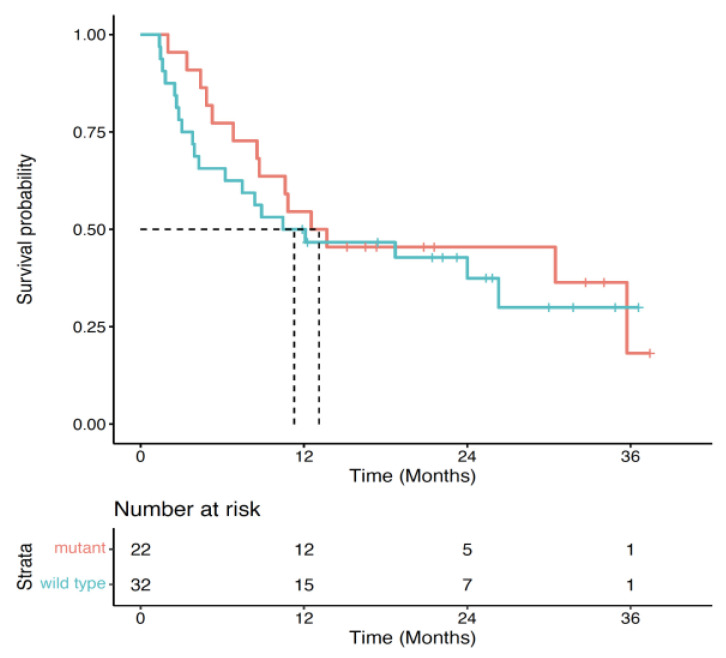
Overall survival by treatment for first-line systemic therapy by *BRAF* status.

In the first-line setting, the 2 year survival was highest for patients that received ipilimumab or ipilimumab plus nivolumab (0.61; 95% CI: 0.50–0.75) or anti-PD-1 (0.54; 95% CI: 0.45–0.65), and lowest for patients that received targeted therapy (0.35; 95% CI: 0.26–0.48) (*p* < 0.001; Figure 3A). Among patients that were tested for a *BRAF* mutation, the 2 year survival probability from first-line treatment was slightly higher for *BRAF*-mutant patients (0.46.; 95% CI: 0.29–0.72) compared to *BRAF*-wildtype patients (0.37; 95% CI: 0.23–0.61) (*p* = 0.57; Figure 4). Two-year survival was highest for patients that received targeted therapy (0.35; 95% CI: 0.26–0.48) and lowest for patients that received ipilimumab or ipilimumab plus nivolumab (0.29; 95% CI: 0.13–0.64) or PD-1 (0.21; 95% CI: 0.9.6–0.46) in the second-line setting (*p* = 0.77). Overall survival did not vary considerably between de novo and recurrent cases for second-line therapy (Table S1). Cancer-specific survival by line and type of therapy is presented in Table S2.

## 4. Discussion

This study reports on real-world treatment patterns and clinical outcomes of metastatic melanoma patients using a large population-based database in Alberta, Canada. We observed that 60.4% of patients initiated systemic therapy, but that only a small proportion of patients initiated second-line therapy (19.1%). Two-year survival probabilities were modest across lines of therapy, especially for second and third-line therapies (first-line: 0.50, second-line: 0.26, third-line: 0.14). In 2017–2018, we found that the majority of patients for whom chart data could be abstracted were tested for *BRAF* mutation status (88.7%). Several Canadian [21,22,23,26,27,28,29,30,31,32,33,34] and international studies [17,18,19,20,35,36,37,38] have reported melanoma treatment patterns and outcomes, but these studies have been limited in their reporting on multiple lines of therapy or on *BRAF* mutation status.

Anti-PD-1 therapies were the most common treatment type overall and within each line of therapy. Targeted therapy was more commonly used in second and third-line therapies compared to first-line. Median time on therapy was highest for anti-PD-1 across all lines of therapy as well. It is unsurprising that anti-PD-1 therapies were the most commonly used, as there have been large shifts to PD-1 therapies since their introduction in 2017, and these therapies have been shown to increase two-year survival [39]. Similar results were reported by Hanna et al., where anti-PD-1 and other newer therapies represented the majority of treatments given in first-line. As a relatively newer therapy, anti-PD-1 therapy use has not been well described in previous Canadian studies but has been discussed as a first-line treatment option growing in use [21,22,35,40].

As described, one-year survival (0.65 for first-line, 0.47 for second-line, and 0.39 for third-line) and two-year survival (0.50 for first-line, 0.26 for second-line, and 0.14 for third-line) was relatively modest across lines of therapy. The highest overall one-year and two-year survival was achieved for Ipilimumab/Ipilimumab + Nivolumab therapies and the lowest survival was observed for patients that received targeted therapy. However, OS reported in this study was higher than in other Canadian and international investigations. In a Canadian study, Hanna et al. reported a one-year survival probability among all patients at approximately 0.45 from the date of first palliative care [22], and Dai et al. reported crude one-year survival probabilities at 0.34 for patients receiving second-line Ipilimumab [33]. In a similarly sized American study, patients on PD-1 inhibitor Pembrolizumab had one-year and two-year overall survival probabilities of 0.61 and 0.48, compared to our reported OS probabilities of 0.72 and 0.54 [19]. Another international study leveraging real-world data from Australia, Italy, Spain, and Germany reported estimates more comparable to ours, with overall one-year and two-year survival at 0.46 and 0.30 for patients receiving Ipilimumab, compared to our estimates of 0.43 and 0.29 [37]. Our results are also comparable to the two largest studies assessing melanoma treatment patterns. A large American study reported a median OS of 18.8 [35] months and 20.7 months [17], which is comparable to our median survival estimates for first-line to third-line therapies.

The majority of patients diagnosed with metastatic melanoma from 2017–2018 were tested for *BRAF* status and almost half of the patients with *BRAF* testing had a mutant *BRAF* test result (41.8%), which is consistent with estimates from large American studies [17,35]. Statistical analyses assessing important sub-group differences by *BRAF* status were not possible due to small cell counts, although we observed that most *BRAF*-wildtype patients were treated with anti-PD-1 therapies, while the majority of *BRAF* mutant patients were treated with targeted therapy in the first-line setting. Some previous Canadian studies have reported on *BRAF* status among melanoma patients [29,32,34]. Two of these studies reported no differences in BRAF status for treatment response [34] or immunotherapy-related adverse events [21]. Two other international studies report younger ages in *BRAF* mutant status patients, which is similar to our findings [20,35]. In addition, similar to our qualitative findings on treatment patterns by BRAF status, a multi-country study found that *BRAF*-mutant patients were more commonly treated with targeted therapy [37].

This investigation has several strengths. To our knowledge, this is one of the first real-world studies of metastatic melanoma in Canada that examined *BRAF* status and three lines of therapy. In addition, we provided updated estimates since the introduction and growth of anti-PD-1 therapies in Canada, while the majority of Canadian studies were conducted using populations from 2015 or earlier. For this study, we leveraged population-based data which captures information on all individuals in the province of Alberta, regardless of treatment center or referral. This utilization of population-based data likely minimized the risk of selection biases and enhanced the generalizability of these results.

The limitations of this investigation should be highlighted. First, since the Alberta Cancer Registry does not capture recurrent cases we used an administrative data algorithm to identify potential recurrent cases. This may have led to some misclassified recurrent cases in this study. Second, we were only able to describe baseline patient characteristics for de novo patients since these characteristics were collected at the time of early stage for recurrent cases and may not reflect characteristics at the time of diagnosis with metastatic melanoma. This study only examined treatment patterns and outcomes of metastatic melanoma patients between 2015 and 2018. The treatment landscape has evolved since this timeframe, particularly, for immunotherapies. Due to the limited sample size, we were unable to examine ipilimumab monotherapy and ipilimumab plus nivolumab as separate groups or stratified by disease severity. Therefore findings with respect to combined groupings should be interpreted with caution. Lastly, statements regarding the comparative efficacy of therapies cannot be made on the basis of these results since this study was descriptive in nature and did not attempt to control for confounding, immortal-time, and other sources of bias.

## 5. Conclusions

We describe treatment patterns and clinical outcomes in a relatively large, population-based cohort in Canada. Our findings are comparable to similar studies conducted in Canada and internationally, with a high patient burden, low overall survival, and a low number of patients progressing to subsequent therapies. Given these findings, we highlight the continued need for effective therapies targeted at metastatic melanoma.

## Figures and Tables

**Table 1:** Baseline and demographic characteristics of de novo metastatic melanoma patients in Alberta, Canada (diagnosed between 2015–2018).

**Variable**	**De Novo** **(*n* = 110)**
**Demographics**	
Age, years (median (IQR))	65.96 (57.31, 76.31)
<65 years (%)	53 (48.2)
≥65 years (%)	57 (51.8)
Male (%)	77 (70.0)
**Socioeconomic Status**	
Urban Residence (%)	91 (82.7)
Neighborhood Annual Household Income (median (IQR))	37,344.46 (31,721.02, 45,489.21)
Quintile Neighborhood Annual Household Income (%)	
Q1 (poorest)	15 (13.6)
Q2	16 (14.5)
Q3	22 (20.0)
Q4	25 (22.7)
Q5 (richest)	32 (29.1)
Proportion of Neighborhood Residents who achieved a Highschool Education or Greater (median (IQR))	0.80 (0.73, 0.84)
Quintile of Neighborhood Education (%)	
Q1 (lowest)	13 (11.8)
Q2	19 (17.3)
Q3	18 (16.4)
Q4	42 (38.2)
Q5 (highest)	18 (16.4)
**Comorbidity**	
Charlson Comorbidity Index (%)	
0	82 (74.5)
1	16 (14.5)
≥2	12 (10.9)
Cardiovascular Disease (%)	11 (10.0)
Diabetes (%)	11 (10.0)
**Metastatic Sites**	
Number of Metastatic Sites at Diagnosis	
1	46 (41.8)
2	28 (25.5)
3	12 (10.9)
≥4	24 (21.8)
Sites of Metastasis at Diagnosis ^1^	
Pulmonary/Pleura	59 (53.6)
Lymph Nodes	46 (41.8)
Brain	31 (28.2)
Hepatic	27 (24.5)
Osseous	23 (20.9)

^1^ Sites of metastases with cell counts < 10 were not reported; Abbreviations: IQR = Interquartile range.

**Table 2:** Baseline and demographic characteristics of metastatic melanoma patients by *BRAF* status in Alberta, Canada (diagnosed between 2017–2018).

Variable	Mutant (*n* = 22)	Wild Type (*n* = 32)	*p*-Value	SMD
Age, years (mean [SD])	61.88 (13.34)	69.34 (13.20)	0.063	0.562
Male (%)	<10 ^1^	21 (65.6)	-	-
Neighborhood Household Income (mean [SD])	39,155.33 (12,149.72)	40,333.23 (16,408.30)	0.776	0.082
Proportion who achieved a Highschool Education or Greater (mean [SD])	0.76 (0.11)	0.76 (0.10)	0.842	0.055

^1^ The number of females was <10. This cell was therefore suppressed; Abbreviations: SD = standard deviations, SMD = standardized mean differences.

## Data Availability

Aggregate-level data presented in this study are available on request from the corresponding author. Individual-level data are not publicly available due to Canadian data privacy laws governing personal health information.

## Supplementary Materials

The following supporting information can be downloaded at: https://www.mdpi.com/article/10.3390/curroncol30040317/s1, Table S1: Overall survival outcomes of metastatic melanoma patients (diagnosed between 2015–2018) by line of therapy stratified by disease type; Table S2: Cancer-specific survival outcomes of metastatic melanoma patients (diagnosed between 2015–2018) by treatment type and line of therapy.
