# Supplementary material for: Understanding Real-World Treatment Patterns and Clinical Outcomes among Metastatic Melanoma Patients in Alberta, Canada
Source: Curr Oncol. 2023 Apr 13;30(4):4166–76. doi: 10.3390/curroncol30040317 (PMC10136717; doi:10.3390/curroncol30040317)
Supplement: Supplementary file 1 [file curroncol-30-00317-s001.zip › curroncol-2267830-supplementary.pdf]

Supplementary Table S1. Overall survival outcomes of metastatic melanoma patients (diagnosed between 2015–2018) by line of therapy stratified by disease type.

Variable	Median Survival (95% CI)	1 Year Survival (95% CI)	2 Year Survival (95% CI)
**1L Systemic therapy**			
De novo	15.29 (8.84–NA)	0.525 (0.424–0.651)	0.445 (0.343–0.577)
Recurrent	25.22 (20.58–38.53)	0.707 (0.645–0.776)	0.522 (0.451–0.604)
**2L Systemic therapy**			
De novo	10.62 (4.41–NA)	0.466 (0.298–0.728)	0.266 (0.122–0.581)
Recurrent	10.16 (5.95–16.37)	0.465 (0.352–0.614)	0.263–0.163–0.425)

Supplementary Table S2. Cancer-specific survival outcomes of metastatic melanoma patients (diagnosed between 2015–2018) by treatment type and line of therapy.

Variable	Median Survival (95% CI)	1 Year Survival (95% CI)	2 Year Survival (95% CI)
**1L Systemic therapy**	28.50 (22.22–NA)	0.689 (0.634–0.749)	0.547 (0.486–0.616)
Ipilimumab/Ipilimumab + Nivolumab	NA (NA–NA)	0.754 (0.654–0.871)	0.666 (0.551–0.804)
PD-1	38.96 (22.22-NA)	0.764 (0.689–0.848)	0.590 (0.498–0.698)
Targeted Therapy	15.29 (9.93–25.22)	0.536 (0.437–0.658)	0.396 (0.299–0.524)
**2L Systemic therapy**	10.62 (7.86–18.94)	0.499 (0.398–0.625)	0.313 (0.215–0.457)
Ipilimumab/Ipilimumab + Nivolumab	8.81 (3.78–NA)	0.429 (0.241–0.762)	0.286 (0.127–0.641)
PD-1	12.07 (4.90–NA)	0.507 (0.361–0.713)	0.245 (0.119–0.502)
Targeted Therapy	19.46 (8.32–NA)	0.564 (0.403–0.789)	0.452 (0.286–0.715)
**3L Systemic therapy**	11.24 (5.65–NA)	0.473 (0.295–0.759)	0.249 (0.101–0.609)
PD-1	12.76 (6.48–NA)	0.533 (0.280–1.000)	0.400 (0.170–0.943)
Targeted Therapy	5.65 (3.75–NA)	0.360 (0.150–0.865)	NA
